# The emerging role of small RNAs in ovule development, a kind of magic

**DOI:** 10.1007/s00497-021-00421-4

**Published:** 2021-06-17

**Authors:** Rosanna Petrella, Mara Cucinotta, Marta A. Mendes, Charles J. Underwood, Lucia Colombo

**Affiliations:** 1 Dipartimento di Bioscienze, Università Degli Studi di Milano, Via Celoria 26, 20133 Milan, Italy; 2 Department of Chromosome Biology, Max Planck Institute for Plant Breeding Research, Carl-von-Linné-Weg 10, 50829 Cologne, Germany

**Keywords:** Ovule, sRNA, Apomixis, RdDM, Female germline, Reproduction

## Abstract

In plants, small RNAs have been recognized as key genetic and epigenetic regulators of development. Small RNAs are usually 20 to 30 nucleotides in length and they control, in a sequence specific manner, the transcriptional or post-transcriptional expression of genes. In this review, we present a comprehensive overview of the most recent findings about the function of small RNAs in ovule development, including megasporogenesis and megagametogenesis, both in sexual and apomictic plants. We discuss recent studies on the role of miRNAs, siRNAs and trans-acting RNAs (ta-siRNAs) in early female germline differentiation. The mechanistic complexity and unique regulatory features are reviewed, and possible directions for future research are provided.

## Ovule development and female germline establishment

**Fig. 1:**
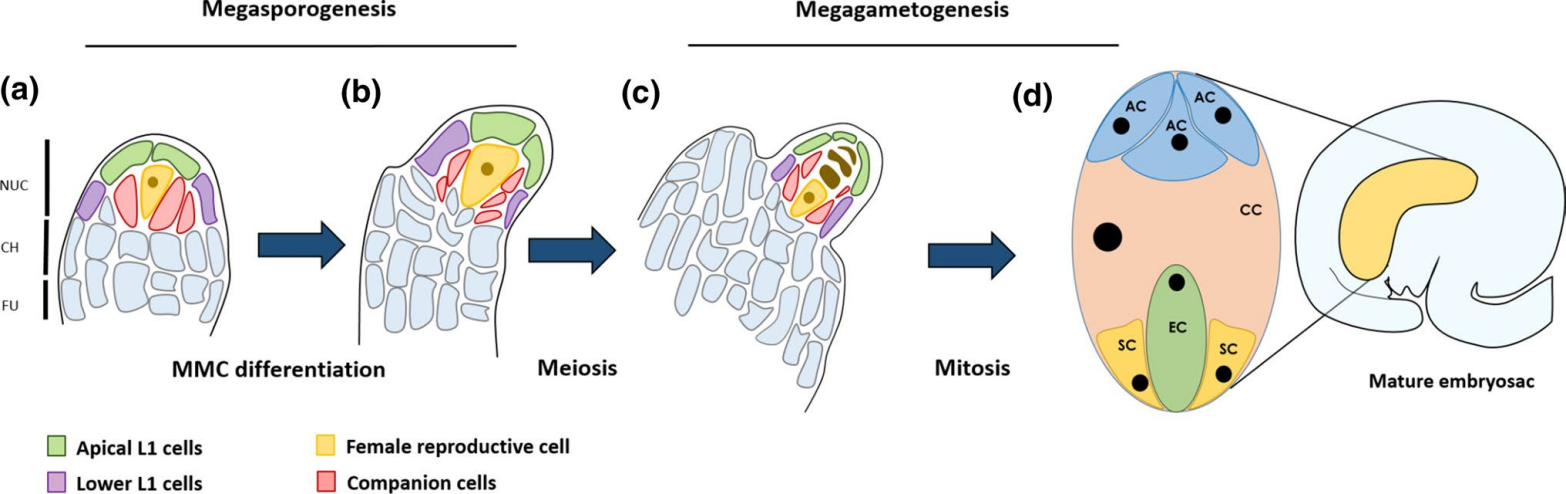
Schematic representation of *Arabidopsis thaliana* ovule development. (**a**) to (**c**) Stages of megasporogenesis. **a** The ovule primordium presents a finger-like shape, wherein different domains can be distinguished: nucellus (nuc), chalaza (ch) and funiculus (fu). Within the nucellus, it is possible to further distinguish singular cell types: green and purple shading highlight the apical and basal epidermis, respectively, also known as L1 layer; dark pink marks the sub-epidermal nucellar cells or companion cells; in yellow, the female germline cell precursor, so called archesporial cell. **b** The female germline precursor differentiates into the megaspore mother cell (MMC); **c** The MMC enters in meiosis, leading to the production of the four haploid megaspores; the most chalazal one survives and the other three undergo programmed cell-death (in brown). The surviving spore is named the functional megaspore. **d** Megagametogenesis starts when the functional megaspore undergoes three rounds of mitotic division. When the mature female gametophyte (or embryo sac) is completely formed, the micropylar pole comprises two synergid cells (sc), one egg cell (ec) and a diploid central cell (cc) whereas the chalazal pole presents three antipodal cells (ac). The inner and outer integuments develop from the chalaza and finally enclose the mature ovule

Ovules are the precursors of seeds and arise from the placenta, a meristematic tissue inside the ovary. Along the proximal–distal axis three regions differentiate: most distal is the nucellus, harboring the female germline; next is the chalaza, from which the integuments arise, and most proximal is the funiculus, which connects the ovule to the maternal tissue. The key steps occurring during ovule development in *Arabidopsis* are summarized in Fig. 1. In the primordium, one of the sub-epidermal cells of the nucellus, the archespore cell, differentiates into the megaspore mother cell (MMC), that undergoes meiosis to form four spores, in a process named megasporogenesis. The three most apical spores degenerate, while the remaining spore, the functional megaspore (FM), enters megagametogenesis. This process consists of three mitotic divisions followed by cellularization to form the embryo sac, the mature female gametophyte (Willemse 1992). The mature female gametophyte, surrounded by the inner and outer integuments, is a polarized structure, consisting of seven cells; we can distinguish two synergid cells, the egg cell, the central cell and three antipodal cells (Drews and Koltunow 2011; Fig. 1). The integuments develop from the chalaza and grow asymmetrically, producing an amphitropous shape (Robinson-Beers et al. 1992). They leave open a minute pore, the micropyle, through which the pollen tube will enter the embryo sac during double fertilization. Consequently, the two released sperm cell nuclei will fuse with the egg cell and the central cell, generating the zygote and the endosperm, respectively, whereas the integuments will develop into the seed coat (Hater et al. 2020).

There is a plethora of studies describing ovule development across evolution (reviewed by Gasser and Skinner 2018). In this review, we will consider the emerging role of small RNAs during ovule development, mainly focusing on the model species *Arabidopsis*.

## Small RNAs pathways: their biogenesis and processing

**Fig. 2:**
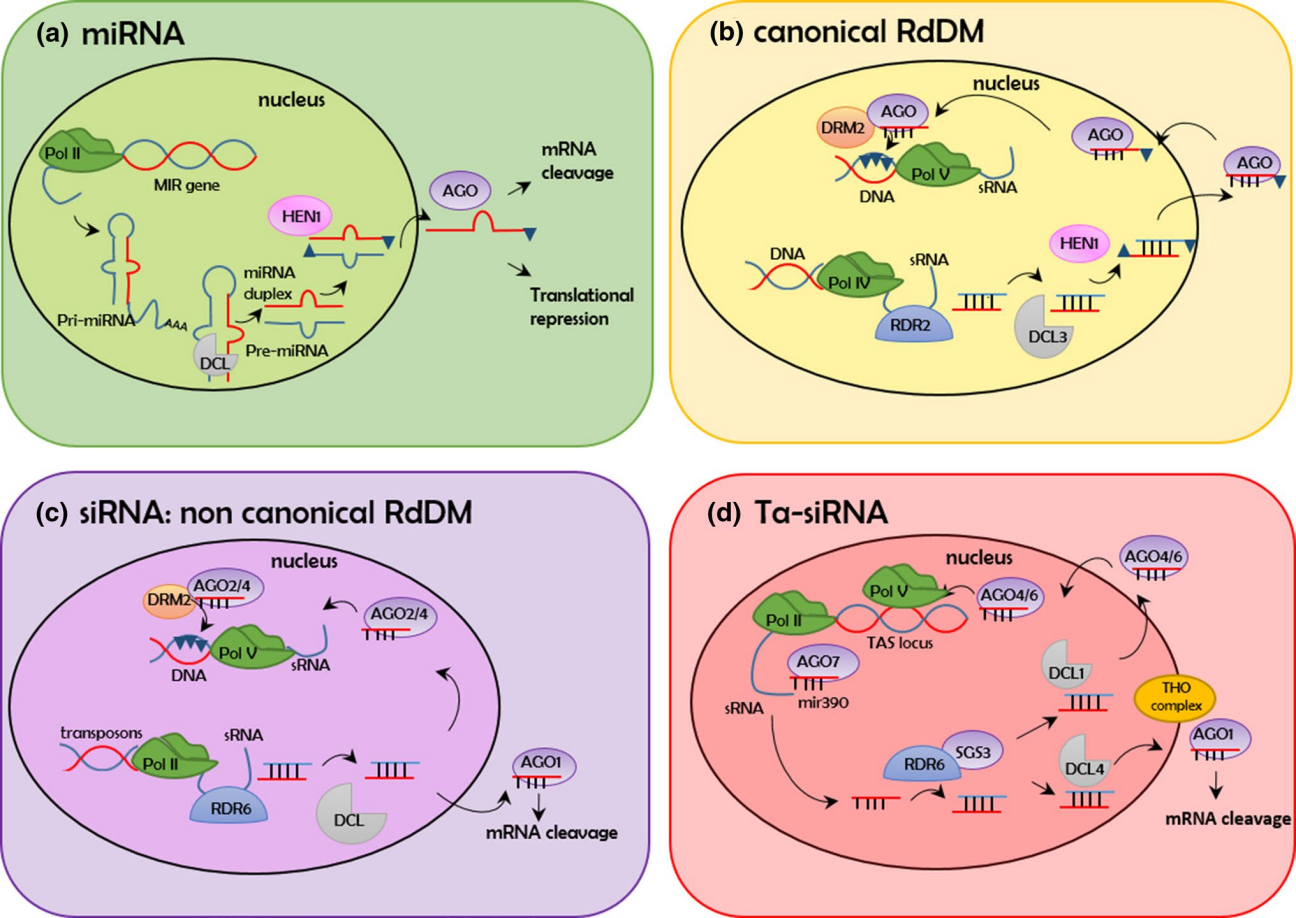
Summary of small RNAs biogenesis and processing. **a** RNA polymerase II (Pol II) is required for the transcription of long primary single stranded miRNA (pri-miRNA); a hairpin structure is then formed by sequence complementarity and the pri-miRNA is cleaved by DICER-LIKE (DCL) proteins, forming the miRNA precursor (pre-miRNA); it is processed by DCL to form miRNA duplexes (Xie et al. 2005). miRNA duplexes are 2ʹ‑O‑methylated at both 3ʹends by HUA ENHANCER 1 (HEN1), a mechanism that protects them from degradation and uridylation; mature miRNA duplexes are recognized and cleaved by the ARGONAUTE (AGO) proteins (Vaucheret 2008); they are successively exported to the cytosol as AGO1:miRNA complexes (Bologna et al. 2018). **b** The canonical RNA-dependent DNA Methylation (RdDM) pathway requires Pol IV to transcribe transposable elements, RNA-DEPENDENT RNA POLYMERASE 2 (RDR2) to produce dsRNAs, DCL3 to process dsRNAs to 24-nucleotide siRNAs, which are stabilized by methylation at their 3′-OH groups by HEN1 and loaded onto AGO4, AGO6, and/or AGO9. During Pol V mediated transcription, the AGO4 recruits DOMAINS REARRANGED METHYLTRANSFERASE 2 (DRM2), that is responsible for de novo methylation at the homologous genomic sites, provoking transcriptional gene silencing (TGS). **c** A slightly different pathway includes the RDR6-dependent RdDM. Young transposons are initially transcribed by Pol II to produce RNAs from transposable elements. Some of these Pol II transcripts can be copied by RDR6 to produce dsRNAs, which are processed by DCL2 and DCL4 into 21–22-nucleotide siRNAs, resulting in AGO1-mediated PTGS of transposon derived RNAs. These dsRNAs can also initiate low levels of de novo DNA methylation, which initiates the canonical RdDM pathway in a manner that is dependent on AGO2, Pol V and DRM2.** d** Trans-acting siRNAs (ta-siRNAs). ta-siRNA biogenesis involves the targeting of a Pol II generated long non-coding *TAS* RNA precursor by miRNA-guided AGO cleavage (miR390-AGO7), followed by the respective synthesis (by RDR6) and stabilization (by SUPPRESSOR OF GENE SILENCING 3; SGS3) of dsRNAs. These dsRNAs are processed by DCL4 and loaded into AGO1 proteins for PTGS, while for non-canonical RdDM they are processed by DCL1, loaded into AGO4/6, can induce DNA methylation at the corresponding *TAS* loci (Matze and Mosher 2014). Ta-siRNAs biogenesis can be further controlled by the THO complex, in particular by TEX1. The THO complex is important to export the ta-siRNAs from the nucleus to the cytosol

A number of small RNA pathways have been reported in plants; while they may differ at the level of biogenesis and functional route of action, they share a single common goal—gene silencing. Gene silencing can occur by inhibiting transcription via DNA cytosine methylation (transcriptional gene silencing, TGS) or by targeting complementary mRNAs for degradation (post-transcriptional gene silencing, PTGS) and finally at the level of translation (translational gene silencing). In plants, small RNAs were first discovered as PTGS factors that act as a cellular defense mechanism against RNA viruses (Hamilton et al. 1999). Subsequently, they were shown to regulate the expression of transposable elements (TGS) and endogenous genes (reviewed by Borges and Martienssen 2015). In Fig. 2, we have schematically described the small RNA processing pathways mentioned in this review as they have been reported to have a role in ovule development.

In brief, miRNAs are 20–24 nucleotides in length and they are encoded by genes, producing a precursor transcript that forms a hairpin structure (pri-miRNA), which is then processed by a DICER-LIKE (DCL) protein, forming the pre-miRNA and successively the mature miRNA duplex; finally, only one strand is loaded into an ARGONAUTE (AGO) protein (reviewed by Borges and Martienssen 2015). In contrast to miRNAs, siRNAs can be produced by diverse routes; the best studied example is represented by siRNA biogenesis, which leads to RNA-directed DNA methylation (RdDM; Matzke and Mosher 2014). In canonical RdDM, a nascent RNA transcript of a transposable element (TE), usually transcribed by the RNA polymerase IV (Pol IV), is targeted by an RNA-dependent RNA polymerase (RDR) to produce a double-stranded RNA, which is then processed by a DCL protein into a siRNAs duplex; successively, only a single 24 nucleotide RNA strand is used by AGO proteins. In RdDM, the AGO proteins can also recruit DOMAINS REARRANGED METHYLTRANSFERASE 2 (DRM2), a DNA methyltransferase, to the transcribed locus to induce DNA cytosine methylation (Zhong et al. 2014). Furthermore, recent findings reported a yet uncharacterized role of AGO1 in the nucleus; here, guided by 21 nucleotide sRNAs, it can active gene transcription in concert with SWI/SNF chromatin remodelling complex (Liu et al. 2018).

Trans-acting siRNAs (ta-siRNAs) are encoded by a class of genes and they act in *trans*, as they are able to regulate target genes that are different from their original loci by PTGS and, in some instances, induce DNA methylation of the *TAS* loci themselves. Ta-siRNA biogenesis involves the targeting of a long *TAS* RNA precursor by miRNA-guided AGO cleavage, followed by the respective synthesis (by RDR6) and stabilization (by SUPPRESSOR OF GENE SILENCING 3; SGS3) of dsRNAs. These dsRNAs are processed by DCL and AGO proteins to form a 21 nucleotide ta-siRNA that can act either through PTGS to silence developmental regulators or TGS, targeting the corresponding TAS loci (Matzke and Mosher 2014).

## Role of RdDM and ta-siRNAs in female germline specification and progression

During ovule development, only one cell of the nucellus differentiates into the MMC. The genetic network controlling MMC specification has been recently reviewed by Pinto et al. (2019). The pre-meiotic ovules of *Arabidopsis* *argonaute 9 (ago9)* mutants have supernumerary MMC-like cells; thus, it has been suggested that AGO9 is required to inhibit the somatic cells surrounding the MMC to acquire a germ cell fate (Olmedo-Monfil et al. 2010; Rodríguez-Leal et al. 2015). AGO9 is reported to primarily act in a non-cell-autonomous manner, as it is preferentially expressed in the somatic cells of the L1 layer of the pre-meiotic ovule. It is worth mentioning that the protein was reported to be transiently and sporadically expressed in the nucleus of the MMC, suggesting a yet uncharacterized role for AGO9 in promoting female germline establishment and progression (Rodríguez-Leal et al. 2015). The ortholog of *AGO9* in *Zea mays, AGO104*, is important for male and female gametophyte development; in fact, the mutant *ago104* is impaired in chromatin condensation during meiosis and subsequently chromosomes fail to segregate properly (Singh et al. 2011). Similar to *AGO9*, also *AGO104* expression is preferentially restricted to the somatic nucellar cell of the ovule primordium. Despite their similar expression domain in the ovule, *AGO104* and *AGO9* seem to act in two different ways since *AGO104* most likely represses somatic fate in germ cells, whereas *AGO9* inhibits germ cell identity in somatic tissue (Olmedo-Monfil et al. 2010; Singh et al. 2011; Rodríguez-Leal et al. 2015; Mendes et al. 2020). AGO9 is preferentially associated with 24 nucleotide siRNAs derived from TEs (Olmedo-Monfil et al. 2010; Havecker et al. 2010). Furthermore, it has been shown that *ago104* mutants have reduced non-CG DNA methylation and increased transcription of centromeric repeats. These observations suggest that AGO9/AGO104 in plants perform similar functions as the animal PIWIs that promote oogenesis by maintenance of transposon silencing in the germline genome (Houwing et al. 2008; Singh et al. 2011).

Similar to the *ago9* mutant, plants lacking the canonical RdDM factors RDR2, DCL3 and Pol IV/Pol V also exhibit a multiple MMC-like cells phenotype (Olmedo-Monfil et al. 2010). Intriguingly, the non-canonical RdDM *rdr6* showed extra numerary MMC-like cells in the nucellus of pre-meiotic ovules (Olmedo-Monfil et al. 2010; Mendes et al. 2020). A similar phenotype has been recently reported for the double mutant in *DRM1* and *DRM2* (Mendes et al. 2020), whose proteins catalyze the DNA methylation of TEs (Law and Jacobsen 2010). All these data suggest that the RdDM pathway is required to confine germ cell identity to a single cell in the ovule. The homologs of *DRM1* and *DRM2* in maize (i.e., *DMT102* and *DMT103*) are both expressed in a restricted portion of the nucellus during megasporogenesis and their loss-of-function leads to the induction of an apomixis-like phenotype (further described in this review), supporting a conserved role for the DNA methylation pathway during female germline formation (Garcia-Aguilar et al. 2010).

Despite the presence of supernumerary MMC-like cells in the nucellus of mutants impaired in RdDM, only one MMC will undergo meiosis (Olmedo-Monfil et al. 2010; Mendes et al. 2020), suggesting that other factors are required to promote germ cell fate in somatic cells. Mendes et al. (2020) investigated the regulatory pathways that act upstream and downstream of RdDM activity and reported that SEEDSTICK (STK), a MADS domain transcription factor that controls ovule identity (Pinyopich et al. 2003; Favaro et al. 2003), directly binds the regulatory region of *AGO9* and *RDR6* to promote their expression solely in the lower cells of the L1 layer. Indeed, the *stk* mutant shows supernumerary MMC-like cells and a reduction in *AGO9* and *RDR6* expression (Mendes et al. 2020). In the nucellus, the RdDM pathway is required to limit *SPOROCYTELESS/NOZZLE (SPL/NZZ)* expression; as a consequence, SPL/NZZ is confined to the L1 cells at the top of the nucellus (Mendes et al. 2020). *SPL/NZZ* is required for germline differentiation, evidenced by the fact that *spl/nzz* mutants fail to form the MMC (Yang et al. 1999; Schiefthaler et al. 1999). Knock-out mutations in RdDM machinery genes (i.e., *AGO9*, *DRM1* and *DRM2*) cause an ectopic expression of *SPL/NZZ*, which is most likely responsible for the formation of the extra numerary MMC-like cells, observed in the mutants described above.

These findings suggest that although the somatic cells surrounding the MMC have somehow the competence to acquire MMC identity, other factors are required to differentiate a fully functional MMC, committed to meiosis (Mendes et al. 2020). Intriguingly, Nonomura et al. (2007) proposed that in *Oryza sativa* there is an excess of archesporial cells in the pre-meiotic ovule but only one undergoes a differentiation process to ultimately form the MMC, most likely lead by the *SPL/NZZ* ortholog (Ren et al. 2018).

**Fig. 3:**
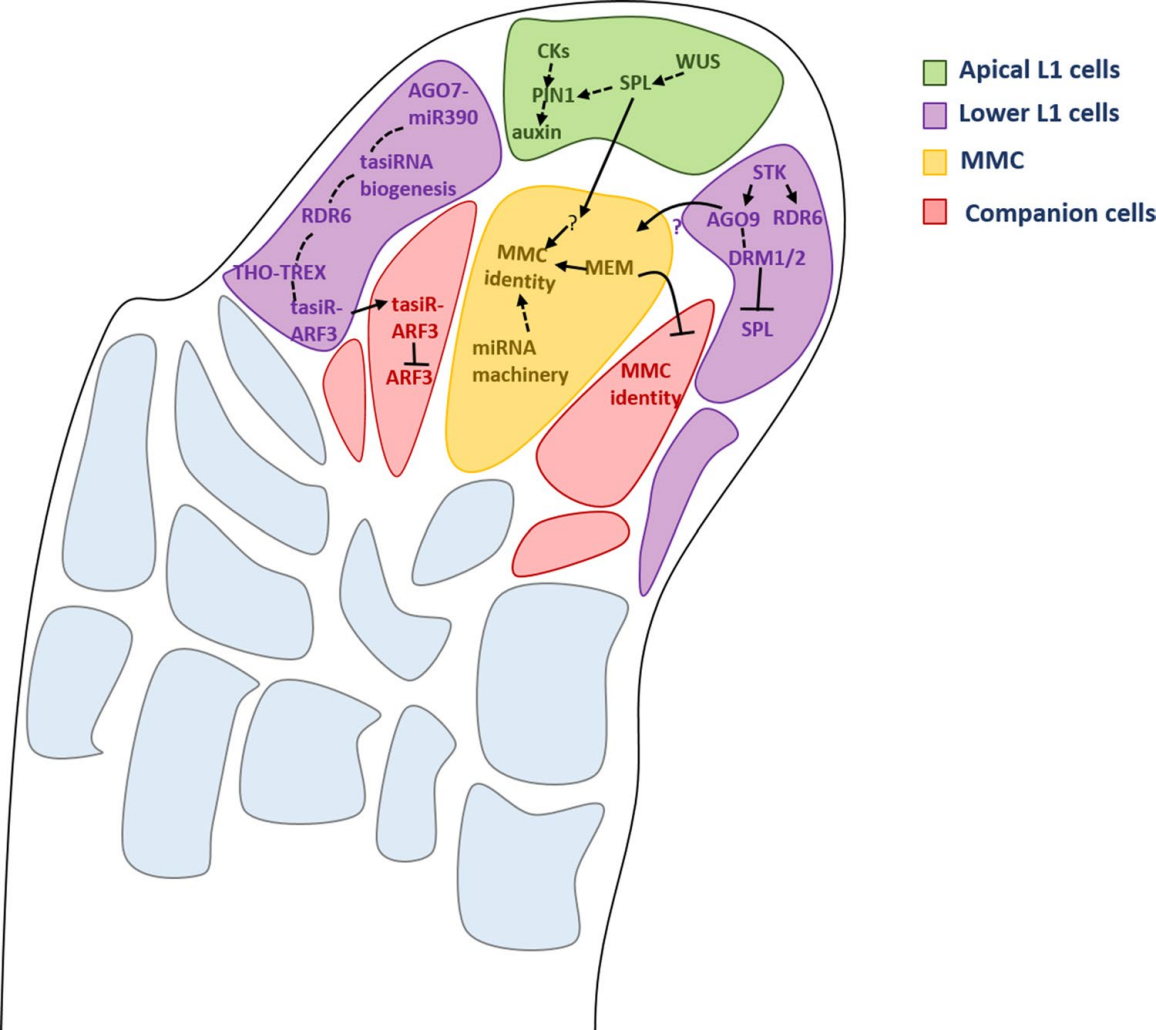
Summary of the key small RNA pathways involved in female germline differentiation. In the *Arabidopsis* ovule the megaspore mother cell (MMC), in yellow, is surrounded by the companion cells (in red) and the L1 layer (in green and purple). The MMC correct differentiation and identity depends on the proper establishment of cell specific small RNA-dependent pathways. The MNEME (MEM), an RNA helicase known to interact with small RNA pathways, is expressed within the MMC; the respective mutant presents multiple MMC-like cells (Gustafson et al. 2010), meaning that its presence is important to restrict MMC cell fate to a single cell. Similarly, several mutants of the RdDM pathway were described as presenting the same *ago9* phenotype (e.g., the *rna-dependent rna polymerase 2* and *6* (*rdr2* and *rdr6*) and the *dicer-like 3* (*dcl3*) (Olmedo- Monfil et al. 2010). The MADS domain transcription factor SEEDSTICK (STK) was shown to directly activate *AGO9* and *RDR6,* and their specific control in the basal L1 cells was shown to be important for a small RNA-dependent methylation pathway, as mutants for *DOMAINS REARRANGED METHYLTRANSFERASES 1* and 2 (*DRM1* and *DRM2*) resulted in multiple MMC-like phenotype as well. This STK-RdDM pathway results in the small RNA silencing (directly or indirectly) of the putative transcription factor SPOROCYTELESS/NZZ (SPL/NZZ), whose expression should be only confined to the apical L1 cells (Mendes et al. 2020). SPL/NZZ and WUSCHEL (WUS) act together in the apical L1 cells to drive the expression of *PIN-FORMED1* (PIN1); its proper localization is further controlled by cytokinins, which are important for the correct auxin flux. SPL/NZZ (and WUS) might send a signal (?) to the hypodermal cell (in yellow), that then acquires MMC identity. Trans-acting short interfering RNA (ta-siRNA) *TAS3* pathway mediated by RDR6 and TEX1 is involved in the repression of AUXIN RESPONSIVE FACTOR 3 (ARF3), a positive regulator of MMC identity

In animals, small RNA silencing pathways have been shown to functionally interact with the Vasa family of RNA helicases in the germline (Gustafson et al. 2010). Despite the fact that no Vasa proteins have been found in the plant kingdom, other RNA helicases might have adopted a similar function. For example, the RNA Helicases MNEM (MEM) has been reported to play an important role during MMC differentiation. The heterozygous *MEM/mem* mutant is indeed characterized by supernumerary MMC-like cells, phenocopying the reported small RNA silencing machinery mutants (Olmedo-Monfil et al. 2010; Schmidt et al. 2011; Mendes et al. 2020). Thus, it had been proposed that MEM might act in combination with the small RNA pathway to restrict the female germline identity to a single cell in the ovule (Schmidt et al. 2011). Notably, the authors reported that *MEM* expression resulted enriched in the MMC, indicating that MMC suppresses the development of additional germline precursors in a non-autonomous fashion. All in all, these findings suggest the requirement of multiple genetic factors involved in restricting germ cell fate to a single cell, the MMC (Fig. 3).

Ta-siRNAs play a fundamental role throughout plant development (Deng et al. 2018); mutants in ta-siRNA biogenesis factors like *sgs3* and *rdr6* showed extra numerary MMC-like cells in the nucellus of pre-meiotic ovules (Olmedo-Monfil et al. 2010; Mendes et al. 2020). Ta-siRNAs belong to a class of endogenous siRNA acting *in trans*, to finely regulate their target genes. Su and collaborators (2017) performed an EMS-based forward genetic screen on the *rdr6* mutant to identify novel genes involved in MMC differentiation. They isolated a mutant, *j66*, that enhances the *rdr6* phenotype, showing extra numerary MMC-like cells in pre-meiotic ovules. Although in the *j66* mutant more cells adopted MMC identity, only one undergoes meiosis to form the functional megaspore (FM). Interestingly, a low percentage of mature ovules show twin embryo sacs, suggesting that the extra MMC might generate an unreduced female gametophyte. Analysis of the *j66* mutant demonstrated that the mutation was located in the *TEX1* gene, which encodes for one of the components of the THO/TREX complex. THO/TREX complex plays an important role in small interfering RNA-dependent processes in plants; in fact, its impairment leads to defects in small RNA biogenesis, export and processing (Francisco-Mangilet et al. 2015). To confirm the role of the THO/TREX complex in germline specification, several THO core complex encoding gene mutants were examined (Masuda et al. 2005; Yelina et al. 2010; Su et al. 2017). Both *hpr1-6* and *tho6* mutants presented extra numerary MMC-like cells in pre-meiotic ovules, suggesting an important function for the THO core complex in MMC differentiation. The THO/TREX complex is involved in ta-siRNA biogenesis by transporting ta-siRNA precursors from the nucleus to the cytoplasm (Jauvion et al. 2010; Yelina et al. 2010). Long non-coding RNAs from the *TAS3* loci are targeted by the miR390-AGO7 complex to trigger tasiR-ARF production. The cleaved transcripts are then stabilized by SGS3 and converted into dsRNA by RDR6 (Kumakura et al. 2009). The ta-siRNA-ARF complex is finally recruited into an AGO1-containing effector complex to repress *AUXIN RESPONSE FACTOR 3 (ARF3)* expression (Fahlgren et al. 2006; Montgomery et al. 2008). The *TAS3* gene is expressed broadly in the ovule, whereas *SGS3* and *AGO7* expression is confined to the epidermis layer, most likely to limit tasiR-ARF biogenesis to the L1 cells in the nucellus. Su et al. (2017; 2020) showed a non-autonomous activity for *ARF3* in female germline specification. In fact, *ARF3* is a positive regulator for MMC identity; the specific expression of *ARF3m* (resistant to regulation by tasiR-ARFs) in the hypodermal cells (i.e., companion cells) is sufficient to trigger the formation of extra MMC-like cells in pre-meiotic ovules, whereas its ectopic expression in the outermost epidermal cell of the L1 layer does not increase the number of MMC per ovule (Su et al. 2020). Thus, the data reported confirm that hypodermal cells have the “potential” to acquire MMC fate. Despite this observation, the extra numerary MMC-like cells do not enter meiosis; hence, further factors are required for the progression of the female germline in those cells. L1 and L2 domains are two specialized cell layers in the nucellus, characterized by distinct transcriptomes (reviewed by Pinto et al. 2019). Therefore, it might be that uncharacterized factors, absent in the L1 layer, are fundamental for complete MMC differentiation and competence to enter meiosis. Su and collaborators (2020) proposed that tasiR-ARF3 can move in two directions: from the epidermal to the hypodermal cell, and from the chalaza to the nucellus, to restrict *ARF3* expression to the chalaza.

To determine the regulatory network downstream of *ARF3* in MMC specification, Su et al. (2020) performed a transcriptome analysis to identify differentially expressed genes (DEGs) in the *pWRKY28*:ARF3m-mCitrine line that specifically drives the expression of ARF3m in the hypodermal cells surrounding the MMC. They pointed out that several factors involved in auxin and cytokinins (CKs) signal transduction pathways were deregulated. The results from Su and collaborators (2017, 2020) unravel a novel role for ARFs in the early step of ovule development, proposing that phytohormones might play an important role in MMC specification and development. The contribution of CKs and auxin in MMC formation has been previously suggested. In fact, *SPL/NZZ* is reported to act downstream of CKs, and to promote the expression of the auxin efflux carrier protein *PIN-FORMED 1 (PIN1)* in the nucellus, required for MMC identity (Bencivenga et al. 2011, 2012). Furthermore, the homeodomain transcription factor *WUSCHEL* (*WUS*; Groß–Hardt and Laux 2003) was suggested to have a role in assuring MMC differentiation by activating *SPL/NZZ* expression in the nucellus; as matter of fact, the *wus* mutant sporadically exhibited ovules without MMC, also shown for *pin1* and *spl/nzz* mutants (Yang et al. 1999; Schiefthaler et al. 1999; Groß–Hardt and Laux 2003; Bencivenga et al. 2011; Bencivenga et al. 2012). Recent findings by Zhao and collaborators (2017) addressed a new role for *WUS* regulation in the megasporogenesis process; indeed, the direct inactivation of the homeodomain factor by Retinoblastoma homolog RBR1 is necessary for the determination of meiotic cell division program in the MMC.

The involvement of diverse factors and genetic networks in MMC differentiation and megasporogenesis intriguingly suggests that different pathways might act in concert to assure the proper establishment and progression of the female germline, through the regulation of hub factors.

## The miRNA machinery plays an important role in megasporogenesis

The microRNA machinery has also been shown to have a role in female germline establishment. It has been reported that mutation in miRNA pathway components affects female germline specification (Oliver et al. 2017). Mutants in genes encoding for components of the different steps of the miRNA pathway have been analyzed: *DCL1* and *HYPONASTIC LEAVES 1 (HYL1),* required for miRNA precursor processing; *HUA ENHANCER 1 (HEN1),* involved in miRNA duplex stabilization; *HASTY (HST),* required in nuclear export of miRNAs and finally *AGO1,* involved in silencing of miRNA targets (reviewed by Wang et al. 2019). Although the mutants do not show a fully penetrant female sterility phenotype, they partially failed to form the MMC (Oliver et al. 2017). Loss of function of miRNA machinery also resulted in the deregulated expression of several genes involved in meiosis and chromatin condensation, many of which could not be explained by direct targeting by miRNAs (Oliver et al. 2017; Wang et al. 2019)*.* Among them, *SWITCH 1 (SWI1)*, a chromatin remodelling gene involved in meiotic recombination and sister chromatid cohesion, *MMS AND UV SENSITIVE 81 (MUS81)*, involved in promoting crossing over (CO) formation, *SMC4A,* which encodes for a protein of the condensin complex, and *TOPOISOMERASE II (TOPII),* which is involved in CO resolution, are predicted targets of miRNAs, according to psRNATarget (Olivier et al. 2015; Dai et al. 2018); they were all overexpressed in the miRNA machinery mutants (Oliver et al. 2017). In particular, *MUS81* was found to be a putative target of miRNA854a-e; miRNA854 has been found in wild-type flowers, but is absent in *dcl1, hyl1* and *hen1* mutants (Arteaga-Vázquez et al. 2006). Therefore, Oliver and colleagues (2017) proposed that the impairment of miRNA machinery leads to a deregulation of key components of meiotic recombination and chromatin condensation in archesporial cells, thus affecting pre-meiotic cell divisions. As a matter of fact, these mutations showed defects in chromatin condensation during first meiotic division and meiosis abortion; in particular, the mutants analyzed (see above) shared several meiotic phenotypes (e.g., decreased number of cells that enter meiosis, increased number of chiasmata, and partial chromosome decondensation from pachytene to metaphase I). Intriguingly, Oliver ﻿﻿and colleagues﻿﻿ (2014) reported a novel role of several AGO proteins during microsporogenesis in the male meiocytes. Despite the observation that impairment in AGO proteins activity does not significantly affect the meiotic process, the authors showed that *ago9-1* mutants were characterized by an higher frequency of chromosome interlocks in mid-late pachytene, while the *ago2-1* mutant had a modest increase in chiasma frequency. Those observations suggest that different classes of AGO protein may be involved in the meiotic process. Given the reported expression of AGO2, AGO5 and AGO9 in the ovules (Olmedo-Monfil et al. 2010; Wuest et al. 2010), it is tempting to speculate that the said proteins might also have a role in meiotic progression in the MMC. All in all, these studies have contributed to shedding light into the role of miRNA machinery during the early phases of germline development (Fig. 3).

## Role of small RNA pathways during megagametogenesis

**Fig. 4:**
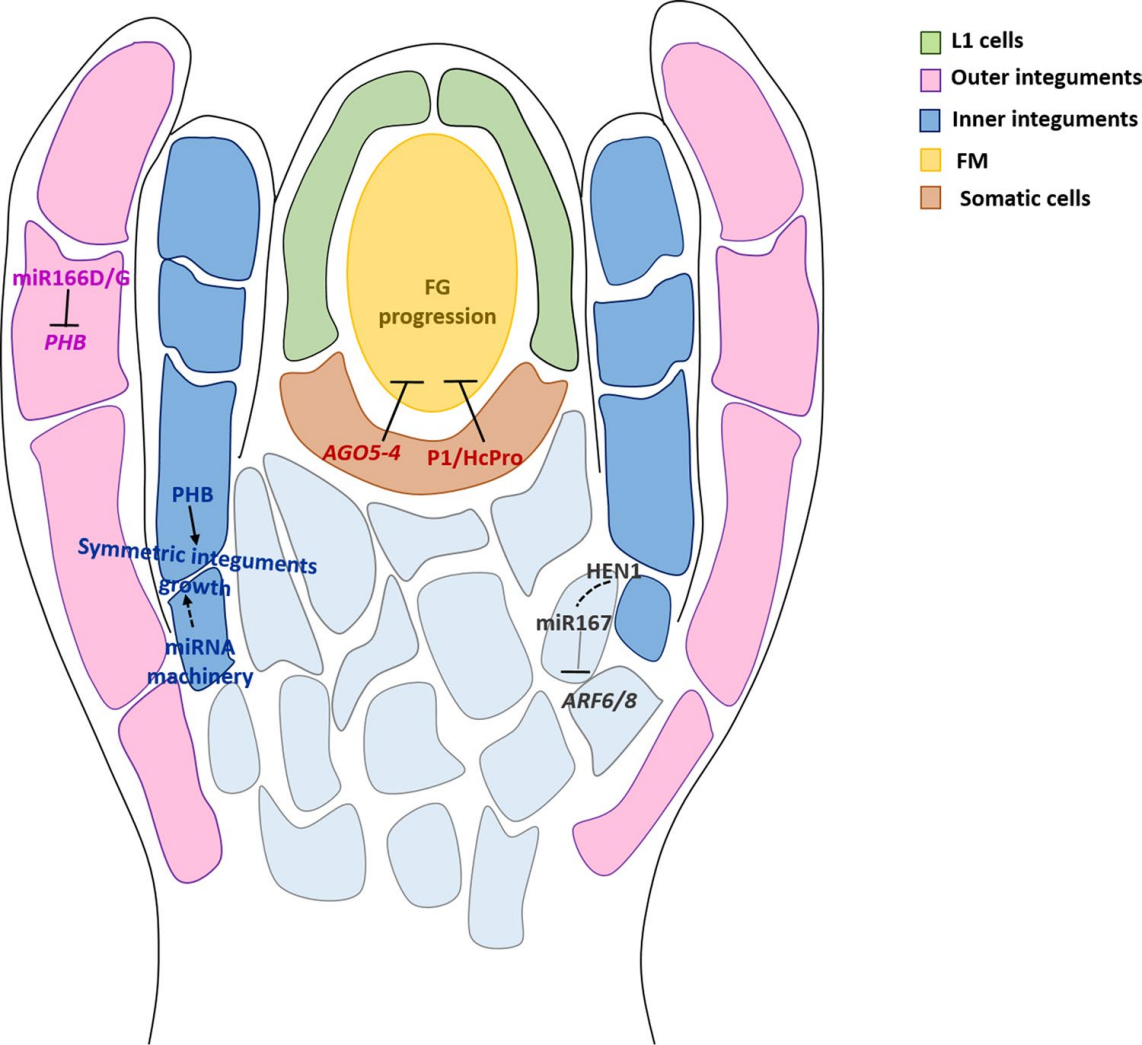
Summary of the key small RNAs pathways implicated in megagametogenesis activation and integuments formation. Upon megasporogenesis, the functional megaspore is formed and megagametogenesis takes place; at the same time inner and outer integuments start to develop and to elongate. Several miRNA pathways have been demonstrated to have an important role in integument growth. miR167 targets AUXIN RESPONSE FACTOR 6 (ARF6) and ARF8, and this regulation is important for the asymmetric integument growth leading to abnormal embryo sac development. A similar situation was encountered in *hen1* mutations; miR165 and miR166 silence the HD-ZIP III family targets, including *PHABULOSA (PHB)*, *PHAVOLUTA (PHV)* and *CORONA (CNA*; Sakaguchi et al. 2012). In fact, the corresponding mutants present defects in inner and outer integuments development. Gametogenesis is impaired in an ARGONAUTE 5 (AGO5) mutant, showing a high percentage of ovule abortion; the predicted AGO5 protein shares a similar protein structure with some viral suppressor proteins, e.g., P1/Hc-Pro and P19, that are known to inhibit miRNA and siRNA functions

At the end of megasporogenesis, three of the four megaspores degenerate, whereas the one located at the chalazal pole, the functional megaspore (FM), will survive and divide mitotically to generate the mature embryo sac. Tucker et al. (2012) reported a small RNA mutant that affects female gametogenesis. They reported that a mutant in the *AGO5* gene was defective in the gametogenesis process. In particular, the mutation generates a semi-dominant AGO5 protein, named AGO5-4, active in somatic cells flanking the functional megaspore and responsible for inhibiting female gametophyte progression. Notably, full knock-out mutation of *ago5* had no effects on female gametophyte development, suggesting that in *ago5-4* are compromised pathway(s) that are not normally targeted by AGO5. The predicted AGO5-4 protein shared a similar protein structure with some viral suppressor proteins, such as P1/Hc-Pro and P19, that inhibit miRNA and siRNA functions (Kasschau et al. 2003). Interestingly, the specific expression of P1/Hc-Pro in the *AGO5* expression domain triggered female gametophyte abortion, proposing the existence of a somatic sRNA pathway that promotes megagametogenesis (Tucker et al. 2012). These results suggested that the progression of megagametogenesis might be somehow dependent on a fine balance of small RNAs in the somatic cells of the ovule (Fig. 4).

The closest rice homolog of AGO5, *MEIOSIS ARRESTED AT LEPTOTENE 1 (MEL1)* is reported to be important in promoting germline development and maintaining female and male germ cell identity; indeed, the mutant is characterized by the disruption of spores formation during meiosis. *MEL1* is specifically expressed in germ cells, suggesting that a cell-autonomous pathway acts to specify germline fate. MEL1 preferentially binds 5′ cytosine of 21 nucleotide phased small interfering RNAs (phasiRNAs) (Komiya et al. 2014). Interestingly, Nonomura and collaborators (2007) reported that the mutation in *MEL1* locus is associated with a significantly reduced H3K9 dimethylation. Since RNAi and AGO proteins are known to play a role in TGS in other systems (Volpe et al. 2002), the authors speculated a relationship between MEL1 function and H3K9 dimethylation. PhasiRNA biogenesis during rice reproductive development is triggered by miR2118; mutation of *miR2118* leads to complete male and female sterility, suggesting an important role for phasiRNAs in rice reproduction (Araki et al. 2020).

Recent studies have further improved our knowledge of the role of small RNA pathways during megagametogenesis. Sprunck et al. (2019) investigated small RNA pathways contributing to egg cell differentiation, by the use of proliferating callus tissue. With this approach, it was possible to successfully identify miRNAs differentially expressed in the egg cell and in other cell types within the female gametophyte, such as synergid cells.

In addition, distribution of small RNAs in egg cells and sperm cells was analyzed in rice (Li et al. 2020a). It has been shown that both egg cells and sperm cells had reduced abundance of miRNAs relative to siRNA and that each gamete expressed a diverse set of miRNAs. Furthermore, Li and collaborators (2020a) reported that in both gametes, patterns of CHH methylation, typically associated with RdDM, were similar to vegetative tissues, although lower in magnitude.

Jullien et al. (2020) elucidated the expression patterns of all *Arabidopsis* *AGO* genes in the mature ovule. They reported accumulation of almost all AGO proteins in the mature egg cell, compared to the other cell types of the embryo sac. All in all, these studies are novel and comprehensive resources for the further study of small RNAs involvement in egg cell differentiation and fertilization in *Arabidopsis* (Sprunck et al. 2019; Jullien et al. 2020).

## miRNAs are of pivotal importance for integuments development

Several players of miRNA machinery as well as different miRNAs have been shown to have important roles in integument growth. The integuments initiate from the chalaza region during the early stage of ovule development and grow in order to enclose and protect the entire gametophyte. It has been reported that a cross-talk between integuments and developing female gametophyte is constantly required for a proper ovule development and female germline progression (reviewed by Bencivenga et al. 2011).

Mutants in *DCL1* presented ovules with severe integuments defects (Robinson-Beers et al. 1992; Lang et al. 1994; Ray et al. 1996; Fukudome et al. 2017); this result indicated that miRNAs could operate during ovule development to regulate a range of genes necessary for proper integuments development (Schauer et al. 2002).

Indeed, after several years, *miR167* and *miR165/166* have been shown to be required for integument growth. The miR167 targets *ARF6* and *ARF8* in both ovule and anthers (Wu et al. 2006). *miR167*-resistant *ARF6 (mARF6)* plants were sterile due to defects in ovules, presenting developmentally arrested integuments (Wu et al. 2006). miR167 is present exclusively in seed plants, suggesting that it could have arisen together with the formation of sporophytic structures that protect the female gametophyte (Wu et al. 2006). It has been reported that miR167a also regulates *ARF6* and *ARF8* expression during early stages of seed development, as the *miR167a* mutant failed to produce normal embryos and endosperm; this phenotype could be suppressed by mutation in either *ARF6* or *ARF8* (Yao et al. 2019).

Recently, two other miRNA-processing proteins HUA1 and HYL1 have been reported to be required for normal integument growth (Wei et al. 2020). HYL1, a dsRNA-binding protein, interacts with DCL1 during the processing of pri-miRNAs (Kurihara et al. 2006), while HEN1 is a O-methyltransferase critical for miRNA biogenesis (Baranauske et al. 2015). Plant miRNAs and siRNAs carry a 2′-O-methyl group on the 3′-terminal nucleotide, a modification introduced by HEN1 that protects small RNAs from enzymatic activities targeting the 3′-OH. In plants with no HEN1 activity, small RNAs accumulate at a lower level and present heterogeneous size (Yu et al. 2005). *hen1-8* and *hyl1-2* ovules failed to grow asymmetric integuments, leading to defective embryo sac development; thus, it compromises pollen tube guidance, causing reduced fertility (Wei et al. 2020). Ectopic expression of *ARF6* and *ARF8* in *hen1-8* ovules was consistent with the reduction of *miRNA167*, whose processing relies on HEN1 (Yu et al. 2010). However, introducing the *arf6 arf8* double mutant did not suppress ovule defects of *hen1-8*, suggesting the involvement of more microRNAs in this process, which may include miR165 and miR166 (Sakaguchi et al. 2012; Hashimoto et al. 2018).

Both miR165 and miR166 have been widely studied for their role in regulating the polarity of lateral organs. In particular, they contribute to the establishment of the abaxial/adaxial patterning of leaf primordia, through the silencing of their *HD-ZIP III* family targets, including *PHABULOSA* (*PHB*), *PHAVOLUTA* (*PHV*) and *CORONA* (*CNA*; Sakaguchi et al. 2012). *PHB, PHV* and *CNA* are expressed adaxially in the inner integument and loss of function of these genes or their overexpression leads to striking defects in integument formation (Kelley et al. 2009). Accordingly, *miR166D* and *MIR166G,* a subset of *miR165/166* genes that are distinct from those acting in root and leaf, are highly expressed in ovule primordia, where they restrict the expression of *PHB* in the apical region of the incipient inner integument (Hashimoto et al. 2018). miR166 shows non-cell-autonomous activity as its activity domain extends toward the ovule apex by a distance of one cell layer compared with the *miR166D/G* expression pattern (Hashimoto et al. 2018). In absence of miR166 function, PHB remains strongly expressed in the chalaza, resulting in compromised formation of the outer integuments. In a similar fashion, the *miR165/166-insensitive phb-1d/* + mutant accumulates *phb-1d* transcripts in both integuments and arrests the outer integument growth (Sieber et al. 2004). Together, these results pointed out the importance of a precise balance between the relative levels of adaxial (HD-ZIP III) and abaxial (miR165/166) activities to support the correct growth of the two integuments (Fig. 4).

## Role of small RNAs in apomictic ovules

Apomixis is asexual reproduction through seeds, a phenomenon that has evolved independently many times among angiosperms, with three families (Asteraceae, Poaceae and Rosaceae) most represented (Carman 1997). In addition, it is consistently associated with hybridity and polyploidy, but its origin and the underlying genetic mechanisms still represent an unsolved problem. There are generally two patterns of apomixis, depending on whether the embryo is developed from the unreduced female gametophyte (gametophytic apomixis) or directly from the nucellar cells (nucellar embryony; Fei et al. 2019). Since apomixis does not naturally occur in major crop species, its introduction into agronomically relevant plants would enable fixation of the complete genome of elite hybrid genotypes, leading to efficient and consistent production of high-quality grains, fruits and vegetables. Notwithstanding the importance of this process and the big effort invested in studying apomictic reproduction, many aspects of this reproductive strategy are yet to be fully understood (Hojsgaard and Hörandl 2019).

As already extensively discussed in the previous sections, several studies have indicated that small RNAs are involved in regulation of MMC specification and megasporogenesis, suggesting that they could be involved in the switch between sexual and asexual reproduction. In fact, *Arabidopsis* (including *ago9*, *rdr6*, *drm1* and *drm2*) and maize (*ago104*, *dmt102* and *dmt103*) mutants have been reported to show features resembling the initial steps of gametophytic apomixis (Olmedo-Monfil et al. 2010; Singh et al. 2011; Tucker et al. 2012; Zhai et al. 2014; Mendes et al. 2020). In order to identify potential pathways that could contribute to apomictic reproduction, several studies in the last decades have focused on high-throughput analysis of small RNAs. For instance, the study of Amiteye et al. (2011) constituted the first extensive insight into the conservation and expression of microRNAs in Boechera. In this genus*,* diplosporous apomeiosis leads to an unreduced embryo sac (Brukhin et al. 2019). In meiotic diplospory, the embryo sac originates from an MMC that undergoes an aberrant meiosis without recombination and chromosome reduction, resulting in the formation of a dyad of unreduced identical megaspores. Working with Boechera has several advantages: it has a relatively small genome, it is the only known diploid genus where apomixis has been described, and *Boechera* spp. are close relatives of the model plant *Arabidopsis thaliana* (Brukhin et al. 2019). Amiteye and collaborators (2011) identified 51 miRNA families in Boechera that are conserved in angiosperms. Among the targets of said miRNAs, the *SQUAMOSA PROMOTER BINDING 11* (*SPL11;* target of miR156/157) has been reported to be up-regulated in the MMC of apomictic micro-dissected ovules. In *Arabidopsis*, *SPL11* is highly expressed in inflorescences and it is associated with the transition from vegetative to reproductive phase (Shikata et al. 2009), but further investigations are required to dissect if SPL11 plays a role in apomictic reproduction.

With a similar approach, the role of miRNA-mRNA interactions has been investigated in the perennial grass *Eragrostis curvula*, providing sRNA libraries from apomictic and sexual genotypes (Garbus et al. 2019). *E. curvula* polyploids are facultative apomicts and reproduction occurs via diplosporous apomixis. Being adapted to sandy-soil and drought conditions, *E. curvula* represents an excellent forage source in semiarid climate regions, including in its native habitat in Africa. The analysis of *Eragrostis curvula* sRNA libraries, obtained from diplospory apomictic and sexual genotypes, lead to the identification of a number of miRNAs, belonging mainly to three families and differentially regulated in the two genetic backgrounds: miR2275 (which triggers the biogenesis of 24-nucleotide phased sRNAs), *miR156* (which targets *SPLs*) and miR8175 (Garbus et al. 2019). The latter, miR8175, targets a TE, that was exclusively expressed in the apomictic accession. Interestingly, in several species, apomixis linked loci are chromosomal regions characterized by chromosomal rearrangements and accumulation of TEs. Associations between apospory and heterochromatic regions of the genome that are rich in retrotransposons raise the intriguing possibility that TEs might play a role in regulating expression of apomixis-related genes (Ochogavia et al., 2011, Kothani et al., 2014). Other two genes, differentially expressed during female reproductive development between apomictic and sexual plants in *E. curvatula,* were *EcAGO104* and *EcDMT102*, encoding for proteins, whose implication in RdDM has already been reported (Garcia-Aguilar et al. 2010; Singh et al. 2011). These data are consistent with a role of said genes in apomixis; however, this provides no causal link as functional studies on mutants have not been carried out yet (Selva et al. 2017).

Initial characterization of small RNA pathways has also been undertaken in some apospory species, such as *Hieracium*, *Paspalum* and *Hypericum spp.* (summarized in Table 1). In aposporic apomixis, the unreduced embryo sacs develop from a nucellar cell adjacent to the MMC (please, see review of Hojsgaard and Hörandl 2019). Hieracium is a genus belonging to the Asteraceae family*,* with the appearance of small annual or perennial herbaceous plants and a typical inflorescence, similar to yellow daisies. Apomictic members of the Hieracium subgenus Pilosella are often polyploids and they are facultative apomicts, exhibiting apospory in combination with autonomous embryo and endosperm formation (Rabiger et al. 2016). In *Hieracium pilosella*, the whole ovary transcriptome from apomictic and mutant apomictic plants, which presented reversion to sexual reproduction, was compared (Rabiger et al. 2016). This study revealed that genes involved in small RNA biogenesis and chromatin silencing were differentially expressed in the two genotypes. Although eleven *Hieracium pilosella AGO* encoding genes were identified, the putative ortholog of *AtAGO9* was not found, despite its role in restricting MMC specification reported in *Arabidopsis* (Olmedo-Monfil et al. 2010; Rabiger et al. 2016; Mendes et al. 2020).

**Table 1:** Selection of high-throughput small RNA analyses performed on ovules and female reproductive tissues

Species	Experimental approaches	References
*Asparagus officinalis*	sRNA-seq, degrad. analysis	Chen et al. (2016)
*Boechera stricta*	in-silico miRNAs ident. and targets pred.	Amiteye et al. (2011)
*Boechera stricta*	sRNA microarray assay and misRNAs pred.	Amiteye et al. (2013)
*Brassica rapa*	ncRNA ident. and description	Byeon et al. (2017)
*Brassica rapa*	sRNA-seq, siRNA analysis, bisulfite sequencing	Grover et al. (2020)
*Chrysanthemum morifolium*	sRNA-seq, miRNA analysis and targets pred.	Zhang et al. (2015)
*Cleistogenes songorica*	sRNA-seq, miRNA analysis and targets pred.	Wu et al. (2018)
*Citrus spp.*	sRNA-seq, miRNA analysis and targets pred.	Long et al. (2016)
*Corylus spp.*	sRNA-seq, miRNA analysis and targets pred.	Liu et al. (2020a)
*Eragrostis curvula*	sRNA-seq, miRNA analysis and targets pred.	Garbus et al. (2019)
*Ginkgo biloba*	sRNA-seq, degrad. analysis, miRNA targets pred.	Liu et al. (2020b)
*Gossypium hirsutum*	sRNA-seq	Abdurakhmonov et al. (2008)
*Gossypium hirsutum*	sRNA-seq, miRNA analysis and targets pred.	Pang et al. 2009)
*Gossypium hirsutum*	sRNA-seq, degrad. analysis, miRNA targets pred.	Liu et al. (2014)
*Gossypium hirsutum*	sRNA-seq, methylC-seq	Song et al. (2015)
*Gossypium hirsutum*	sRNA-seq, miRNA analysis and targets pred.	Xie et al. (2015)
*Gossypium hirsutum*	sRNA-seq, miRNA analysis and targets pred.	Zhao et al. (2019)
*Hieracium spp.*	sRNA-seq	Rabiger et al. (2016)
*Hypericum perforatum*	in-silico miRNAs ident./targets pred.	Galla et al. (2013)
*Oryza sativa (autotetraploid)*	sRNA-seq, miRNA targets pred.	Li et al. (2017)
*Oryza sativa*	sRNA-seq, miRNA targets pred.	Yang et al. (2017)
*Oryza sativa*	sRNA-seq, miRNA targets pred.	Wu et al. (2017)
*Oryza sativa*	sRNA-seq, methylC-seq	Li et al. (2020a)
*Oryza sativa*	ssRNA-seq and lncRNA ident.	Liu et al. (2019)
*Oryza sativa (autotetraploid)*	lncRNAs ident.	Li et al. (2020b)
*Paspalum notatum*	sRNA-seq, miRNA analysis and targets pred.	Ortiz et al. (2019)
*Pinus taeda*	miRNA detection by RT-PCR	Oh et al. (2008)
*Punica granatum*	sRNA-seq, miRNA analysis and targets pred.	Chen et al. (2020)

degrad. = degradome; pred. = prediction; ident. = identification

New data related to apospory comes from studies in *Paspalum notatum,* an important forage crop native to South America, which includes sexual diploid and apomictic polyploid biotypes. Ortiz et al. (2019) identified in *Paspalum notatum* fifty-six clusters of miRNAs differentially represented in apomictic and sexual plants (up-regulated in apomictic), by combining available transcriptomic resources with small RNA-seq profiling. Interestingly, examination of the potential targets of these miRNAs pointed out an enrichment for genes involved in auxin pathways, from metabolism to signaling. For instance, miR160 and miR167, which regulate *ARFs* expression, were differentially expressed in apomictic plants, suggesting that auxin network might be perturbed during ovule development in *P. notatum* biotypes (Ortiz et al. 2019). Moreover, a previous work in *P. notatum*, identified a long non-coding RNA (named *PN_LNC_N13*), expressed only in natural apomictic tetraploid genotypes and not in artificially tetraploidized sexual genotypes (Laspina et al. 2008; Ochogavia et al. 2018). The sequence of the putative target of *PN_LNC_N13* was used to identify the most likely *Arabidopsis* orthologs and the best match was *ARABIDOPSIS RESPONSE REGULATOR 9 (ARR9)*, involved in cytokinins response (Ochogavia et al. 2018).

Finally, small RNA-seq analysis has been performed also in citrus, one of the most commercially relevant fruit trees worldwide. Citrus is characterized by a nucellar embryony type of apomixis, where asexual embryos initiate directly from unreduced, somatic, nucellar cells surrounding the embryo sac. From a genome-wide association study (GWAS), a candidate gene for citrus polyembryony has been proposed (Wang et al. 2017). In addition, Long et al. (2016) identified 150 miRNAs in ovules immediately prior to and after nucellar embryony initiation, and among them miR23-5p showed negative correlation in expression with their target genes.

## Beyond *Arabidopsis*: the roles of small RNA pathways in ovule development in diverse plant species

Knowledge about the role of small RNAs in ovule and female gametophyte development in other species is still expanding. Several experimental approaches, such as sRNA and lncRNA sequencing, degradome analysis, and target prediction, were performed in ovule tissue samples of several flowering plant species other than *Arabidopsis*, as summarized in Table 1. These studies include ovule samples from agronomically important species including rice, cotton and *Brassica rapa*. Outside of angiosperms, the role of sRNAs in ovule development is underexplored. In gymnosperms, despite recent progress on sRNA profiles in male gametophytes, the contribution of sRNAs to ovule development remains to be understood. In fact, several studies revealed the presence of a pool of 24 nucleotide sRNAs in pollen, while only 21 nucleotide sRNAs have previously been detected in needles (Dolgosheina et al. 2008; Nakamura et al. 2019). More widely, it will be interesting to understand the role of small RNAs in female gametogenesis in the emerging model species *Marchantia polymorpha* (a liverwort) and *Physcomitrella patens* (a moss; Coruh et al. 2015; Bowman et al. 2017).

Several comprehensive analyses of sRNA expression in ovule development have been performed in rice. By including several stages of ovule development in a single rice cultivar, Wu et al. (2017) reported expression of 486 known miRNAs, and 204 novel miRNAs; their targets were then identified. Furthermore, integrated analysis with ovule transcriptome data enabled screening of possible miRNA targets that showed coherent expression levels with miRNAs in ovules (Wu et al. 2017). A similar approach was used to investigate the potential regulatory effects of miRNAs and lncRNAs on rice female gametophyte abortion, comparing the miRNA transcriptome in ovules of a high frequency female-sterile line (*fsv1*) and a wild-type line (Gui 99) during ovule development (Yang et al. 2017; Liu et al. 2019). These studies identified 100 known miRNAs and around 500 lncRNAs that were significantly differentially expressed in *fsv1* compared to the wild-type (i.e., Gui 99); thus, it provides a pool of ncRNAs with a potential role in rice female gametophyte (Yang et al. 2017). One of the major barriers in commercial application of rice polyploids is their low fertility. It has been suggested that differential expression patterns of small RNAs during embryo sac development in autotetraploid rice might be associated with sterility (Li et al. 2017). Among the differentially expressed miRNAs, two of them were associated with female meiosis. Interestingly, their putative targets *MINICHROMOSOME MAINTENANCE FAMILY 9 (MCM9) * and *REPLICATION PROTEIN A 2C (RPA2C)* are known to be meiosis-related genes.

Furthermore, Li et al. (2017, 2020b) identified 24 nucleotide TE-derived siRNAs and lncRNAs, specifically expressed in autotetraploid rice ovules at MMC stage. The expression levels of several genes may change under the influence of TE derived siRNA triggered methylation, particularly at the crucial stages of female meiosis, which explained more than 95% up-regulated TE-derived siRNAs during the embryo sac development in autotetraploid rice (Li et al. 2017). Overall, the list of differentially expressed lncRNA during meiosis in rice ovaries will be beneficial for future studies in plant reproduction, in order to overcome sterility of polyploid rice.

RdDM mediated CHH hypermethylation has been reported in *Gossypium hirsutum* (cotton) ovules and ovule-derived cotton fibers (Song et al. 2015). In cotton ovules CHH hypermethylation is found in 5′ of genes, while in ovule-derived cotton fibers this hypermethylation is enhanced, and CHH hypermethylation is also reported to be on TEs (Song et al. 2015). Genes preferentially expressed in ovules presented 5′ methylation, while this is not the case for genes preferentially expressed in leaves. Treatment of in vitro cultured cotton ovules with a DNA methyltransferase inhibitor, 5-aza-deoxycytidine, leads to reduced cotton fiber length, suggesting that DNA methylation might play a role in cotton fiber development. In the future, the precise modulation of RdDM in cotton ovules and developing cotton fibers would be required to show a causal link between them.

An important role for small RNAs and RdDM has also been established in the crop species *Brassica rapa* (Grover et al. 2018; 2020). Through conventional hybridization, it has been shown that high rates of seed abortion are found from RdDM mutant mothers, but not from RdDM mutant fathers (Grover et al. 2018). This analysis showed that RdDM activity is required in *B. rapa* maternal sporophytic tissue, and not in the female gametophyte for successful seed development (Grover et al. 2018). Small RNAs are highly abundant during female reproductive development in *B. rapa*, and the majority of small RNAs originate from a small number of loci (Grover et al. 2020): these siren siRNAs are present in ovules, seed coats, embryos and endosperm (Grover et al. 2020). RDR2 is essential for the production of siren siRNAs in the maternal sporophyte and the maternal genotype also dictates whether they accumulate in later tissues, suggesting the deposition of maternal siRNAs is important for normal seed development in *B. rapa*.

## Conclusion and future perspectives

Since the discovery of small RNAs in plants (Hamilton and Balucombe 1999), the diversity of plant small RNA biogenesis and function has been gradually revealed, yet the full complexity and inter-relatedness of the pathways is still being explored (Cuerda-Gil and Slotkin 2016). Here, we focused on the role of small RNAs during ovule development, where they appear to play a role in germline specification, megagametogenesis and integument development. Despite significant progress on this topic in recent years, the combination of genetic redundancy of small RNA biogenesis and effector pathways, and the embryo lethality of some *Arabidopsis* small RNA mutants mean that further genetic dissection will likely remain a challenge.

In wild-type sexual species, the number of MMCs within a single ovule is restricted to one. In various *Arabidopsis* small RNA mutants, additional MMC-like cells are observed. The classic RdDM DNA methyltransferase double mutant *drm1 drm2* also has additional MMCs (Mendes et al. 2020). Intriguingly, RDR6 was initially found to be an important part of the co-suppression pathway in the plant immune system (Dalmay et al. 2000; Mourrain et al. 2000). Later, it was shown to play a key role in ta-siRNA biosynthesis (Yoshikawa et al. 2005) and a minor role in RdDM in somatic tissues (Stroud et al. 2013). RDR6 is clearly a “pleiotropic actor” in small RNA biogenesis and exemplifies the complexity of plant small RNA pathways; also, it suggests that attributing labels of distinct pathways to genes is sometimes misleading. Within the RdDM field, discussion continues over the role of “canonical” and “non-canonical RdDM” pathways that may occur in parallel within a single cell or play a specific role in specific cell types (Cuerda-Gil and Slotkin 2016). In the future, it will be interesting to further explore whether the small RNA mutants that give rise to additional MMCs are due to separate small RNA pathways (“canonical” RdDM and ta-siRNAs) or if they could contribute to a “non-canonical” RdDM pathway. Interestingly, Su and collaborators (2017) showed that mutation in *tex1* enhanced the *rdr6* phenotype, suggesting that two parallel pathways might synergically act in female germline fate. Highlighting the complexity of small RNA biology within ovules, all ten known AGO proteins have been detected during female reproduction in *Arabidopsis* (Jullien et al. 2020). Further to this, it has already been shown that AGO4, AGO6 and AGO8 may also contribute to MMC number specification; interestingly, in *ago4 ago9* double mutants *AGO6* expression is increased to likely compensate for lack of *AGO4* and *AGO9* (Hernández-Lagana et al. 2016)*.* The use of cell type specific approaches, including scRNA-seq and conditional CRISPR/Cas9 genome editing, may represent the best route to furthering our understanding of MMC specification by small RNA pathways.

To date, most functional studies of small RNA pathways in ovule development have focused on *Arabidopsis*, along with notable contributions in rice and maize. In numerous other species, ovule enriched small RNAs have been characterized by next generation sequencing (summarized in Table 1) and have therefore been largely descriptive. In future, we expect that functional genetic studies in diverse plant species, powered by CRISPR/Cas9 gene editing and using an evolutionary developmental biology approach, will enrich our understanding of the role of small RNAs in ovule development.
